# Spectral Characteristics, In Silico Perspectives, Density Functional Theory (DFT), and Therapeutic Potential of Green-Extracted Phycocyanin from *Spirulina*

**DOI:** 10.3390/ijms25179170

**Published:** 2024-08-23

**Authors:** Velichka Andonova, Krastena Nikolova, Ivelin Iliev, Svetlana Georgieva, Nadezhda Petkova, Mehran Feizi-Dehnayebi, Stoyanka Nikolova, Anelia Gerasimova

**Affiliations:** 1 Department of Pharmaceutical Technologies, Faculty of Pharmacy, Medical University of Varna, 9002 Varna, Bulgaria; 2 Department of Physics and Biophysics, Faculty of Pharmacy, Medical University of Varna, 9002 Varna, Bulgaria; 3 Department of Pharmaceutical Chemistry, Faculty of Pharmacy, Medical University of Varna, 9002 Varna, Bulgaria; ivelin.iliev@mu-varna.bg (I.I.);; 4 Department of Organic Chemistry and Inorganic Chemistry, University of Food Technologies, 4002 Plovdiv, Bulgaria; nadezhda_petkova@uft-plovdiv.bg; 5 Department of Organic Chemistry, Faculty of Chemistry, Alzahra University, Tehran P.O. Box 19938-93973, Iran; m.feizi@alzahra.ac.ir; 6 Department of Organic Chemistry, Faculty of Chemistry, University of Plovdiv Paisii Hilendarski, 4000 Plovdiv, Bulgaria; tanya@uni-plovdiv.bg; 7 Department of Chemistry, Faculty of Pharmacy, Medical University of Varna, 9002 Varna, Bulgaria; anelia.gerasimova@mu-varna.bg

**Keywords:** *Spirulina*, phycocyanin, FTIR spectra, NMR spectra, DFT, pharmacokinetics, metabolism, toxicity

## Abstract

Phycocyanin (PC) is a naturally occurring green pigment in *Spirulina.* It was extracted by ultrasonic extraction using green technology, and its structure was studied using IR- and NMR-spectroscopy. Spectral data confirmed the PC structure. This study also involves an in silico assessment of the diverse applications of green pigment PC. Utilizing QSAR, PreADME/T, SwissADME, and Pro-Tox, this study explores the safety profile, pharmacokinetics, and potential targets of PC. QSAR analysis reveals a favorable safety profile, with the parent structure and most metabolites showing no binding to DNA or proteins. PreADME/T indicates low skin permeability, excellent intestinal absorption, and medium permeability, supporting oral administration. Distribution analysis suggests moderate plasma protein binding and cautious blood–brain barrier permeability, guiding formulation strategies. Metabolism assessments highlight interactions with key cytochrome P450 enzymes, influencing drug interactions. Target prediction analysis unveils potential targets, suggesting diverse therapeutic effects, including cardiovascular benefits, anti-inflammatory activities, neuroprotection, and immune modulation. Based on the in silico analysis, PC holds promise for various applications due to its safety, bioavailability, and potential therapeutic benefits. Experimental validation is crucial to elucidate precise molecular mechanisms, ensuring safe and effective utilization in therapeutic and dietary contexts. DFT calculations, including geometry optimization, MEP analysis, HOMO-LUMO energy surface, and quantum reactivity parameters of the PC compound, were obtained using the B3LYP/6–311G(d,p) level. This integrated approach contributes to a comprehensive understanding of PC’s pharmacological profile and informs future research directions.

## 1. Introduction

Phycocyanin (PC), a vibrant blue pigment, is a naturally occurring compound primarily found in cyanobacteria and select algae. The name “phycocyanin” stems from the Greek words “phyco” (algae) and “cyanos” (blue), aptly capturing its origin and characteristic color. This water-soluble pigment belongs to the phycobiliprotein family and plays a pivotal role in light absorption during photosynthesis, contributing to the energy conversion process in these photosynthetic organisms [1].

Cyanobacteria, commonly called blue–green algae, represent a significant source of PC [2,3]. These microscopic organisms thrive in diverse aquatic environments, from freshwater ecosystems to marine habitats. PC is a vital light-harvesting pigment in cyanobacteria, allowing them to absorb light energy efficiently for photosynthetic processes. In addition to cyanobacteria, certain types of algae, particularly those belonging to the red and blue-green algae, also contain notable amounts of PC. Algal species such as *Spirulina* and Aphanizomenon flos-aquae are known for their high PC content and are cultivated for various applications, including the extraction of PC for commercial purposes [4,5].

Phycocyanin has a similar structural motif to all phycobiliproteins [6]. The structure is assembled from phycobiliprotein monomers, which are heterodimers made up of α and β subunits and the corresponding chromophores connected by thioether bonds.

PC exists in three forms: C-PC, derived from cyanobacteria; R-PC, derived from red algae; and R-PC II, derived from *Synechococcus* species [7,8].

**Figure 1:**
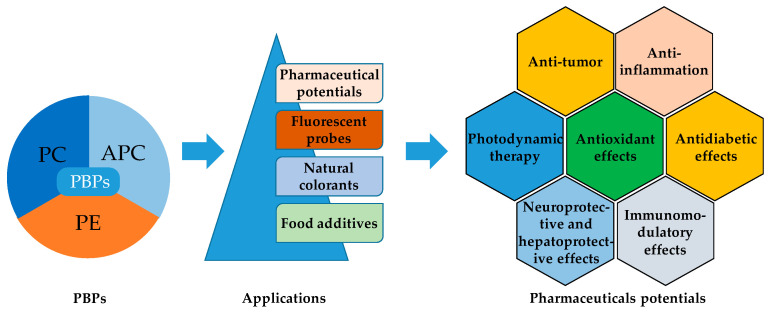
Application as food additives, natural colorants, and fluorescent probes, and the pharmaceutical potentials of PBPs (APC, PC and PE) [8].

C-PC possesses antioxidant and anti-inflammatory properties, as well as neuroprotective, hepatoprotective, and anti-cancer effects (Figure 1) [9,10]. Red macroalgae *Porphyra* spp. (R-PC) is a rich source of proteins, vitamins, minerals, and antioxidants [11].

As a water-soluble phycobiliprotein, PC comprises a polypeptide chain, and chromophores called phycocyanobilins have a molecular mass of about 220 kDa. The polypeptide chain spans alpha (α) and beta (β) subunits with molecular masses between 18 and 20 kDa [12]. The two subunits form monomers that organize into trimers and then into hexamers. Thioether linkages between cysteine residues and chromophores contribute to the stability of PC. Essential amino acids such as serine, histidine, and aspartic acid further support the overall structure. This molecular configuration is critical to understanding PC’s functional properties in various applications, including food colors, nutritional supplements, and biomedical research.

PC prevents inflammation [13,14,15,16,17] due to the inhibition of cyclooxygenase-1 (COX-1) and cyclooxygenase-2 (COX-2) [18]. PC counteracts lipid peroxidation induced by the trichloromethyl radical (•CCl_3_) obtained during the biotransformation in carbon tetrachloride (CCl_4_) liver. PC prevents oxidative damage and reduces lipid peroxidation, assessed by peroxidation potential. In addition, PC induces a decrease in antioxidant enzymes such as GPx, catalase (CAT), glutathione reductase (GR), and glutathione-S-transferase (GST) [19,20].

*Spirulina* is well-known for its high concentration of antioxidants, phytonutrients, probiotics, and nutraceuticals [5,14,18,20,21].

Reactive oxygen species and free radical overproduction are significant contributors to several diseases, including cancer, neurological conditions, heart disease, diabetes, and organ damage [22]. *Spirulina* treatment significantly reduces oxidative stress in people and animals [23,24,25,26].

As a dietary supplement, *Spirulina* can be used to treat various neurological conditions, including Parkinson’s, Alzheimer’s, and Huntington’s disease, and to prevent diseases linked to free radicals [27]. The tetrapyrrolic constituents significantly slow the growth of pancreatic cancer. The high antioxidant activity of *Spirulina,* its ability to limit the formation of ROS by mitochondria, and the resulting changes in intracellular redox status are at least partially responsible for these effects [28], suggesting a potential chemopreventive function.

The toxicity assessment of PC is imperative to ensuring the safety of its applications in food coloring, pharmaceuticals, and dietary supplements. Humans consume PC directly or indirectly as a natural blue food coloring agent, necessitating a comprehensive evaluation of its safety profile to adhere to regulatory standards set by authorities such as the Food and Drug Administration (FDA) and European Food Safety Authority (EFSA). Additionally, its inclusion in dietary supplements for potential health benefits requires a thorough assessment to ascertain safety, considering antioxidant, anti-inflammatory, and immune-boosting properties.

PC is explored in biomedical and pharmaceutical applications for therapeutic effects, such as antioxidant properties, anti-inflammatory activity, neuroprotective effects [29], and immunomodulatory activity [30]. Toxicity assessment is also crucial before advancing to clinical use. The assessment addresses potential carcinogenicity, genotoxicity, and cell damage, ensuring the safety of consumers. Beyond compliance, toxicity assessments contribute to risk mitigation, fostering public trust by transparently demonstrating the PC’s safety in alignment with consumer demands for natural ingredients.

The present study aimed to characterize the structure of green-extracted PC from *Spirulina* and investigate in silico the pigment’s properties for developing functional foods, nutritional supplements, and medicinal products.

## 2. Results and Discussion

Using nuclear magnetic resonance and infrared spectra can help understand and improve the molecular interactions of substances obtained as secondary metabolites based on the in silico study of PC characteristics. The studies above will provide an opportunity to improve the molecular structure of novel pharmaceutical items to boost their bioavailability and activity.

### 2.1. Spectral Study of the PC’s Structure

#### 2.1.1. FTIR Spectrum of PC

**Figure 2:**
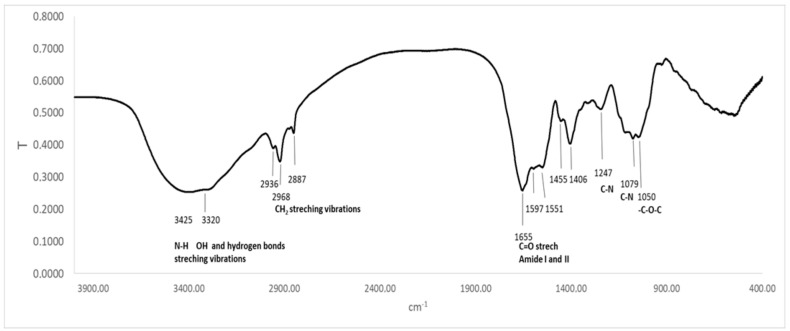
FTIR spectrum of PC.

The FTIR spectrum of PC showed a broad band between 3500 cm^−1^ and 3100 cm^−1^, which can be assigned to O–H stretching vibrations, N–H extension vibration, and the intermolecular H bond. The band at 3425 cm^−1^ is due to νs (N-H), typical for secondary amines. The band at 2936 cm^−1^ could be assigned to ν(C–H) from CH_2_ groups. The bands located at 2968 cm^−1^ and 2936 cm^−1^ were attributed to asymmetric deformations in CH_2_ and C–H stretching. The asymmetric stretching vibration of the carboxylate group appears at 1620–1598 cm^−1^. The band at 1655 cm^−1^ can be assigned to the stretching vibrations of C=C, -C=O, or C=N in primary or secondary amines. The band at 1597 cm^−1^ is due to ν(C=N) in the pyrrole ring. The band at 1554 cm^−1^ is assigned to N-H stretching vibration, while the bands at 1247 and 1079 cm^−1^ were due to C-N stretching vibration. The band at 1050 cm^−1^ is due to C-O-C. The aromatic rings of PC are also in wavenumber intervals from 1500 to 160 cm^−1^. The band at 1455 cm^−1^ was due to δCH_3_ and CH_2_. A similar observation was reported by Kneip, C et al. [31,32,33,34,35,36]. Qiao et al. found bands at 1660 cm^−1^ and 1530 cm^−1^ in PC and PC complexes, attributed to the amide I and amide II groups [33]. In their spectra, the absorption band stands at 1642 cm^−1^, caused by the -C=O stretching vibration or N-H bending vibration, while the absorption peak at 1536 cm^−1^ was caused by the N-H stretching vibration. The FTIR spectrum of PC is shown in Figure 2.

Moreover, according to Al-Malki [37], a PC spectrum possessed transmittance maxima at 1652 cm^−1^ and a characteristic band (2100–3700 cm^−1^), which is indicated mainly from -COO, -CO, and conjugated double bonds.

Garcia-Pliego et al. [38] also observed some typical bands in PC spectra, such as C–H wagging vibration in the region of 1446 and 1418 cm^−1^, as well as C–O stretching from 1100 to 1000 cm^−1^. All these findings approve the structure of PC.

The FTIR spectral data were confirmed by the NMR analysis.

#### 2.1.2. NMR Spectra of PC

**Figure 3:**
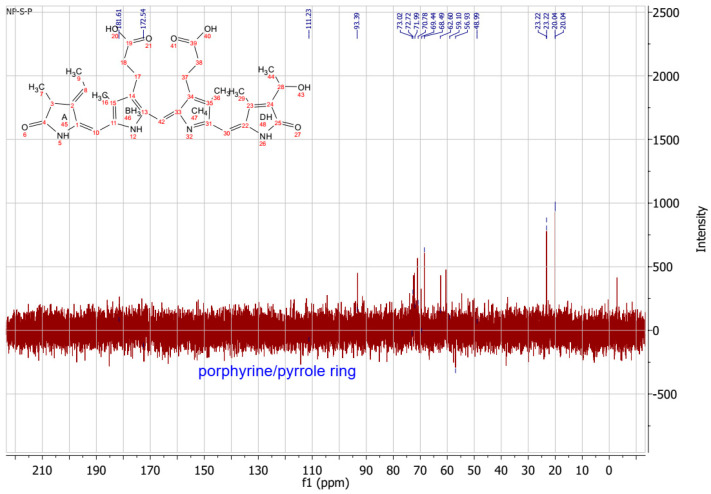
^13^C-NMR of PC.

As previously described by Song et al. [39], chemical shifts that were arranged into three well-separated ranges in 13C NMR spectra were found: aliphatic carbons appearing between 0 and 40 ppm, aromatic carbons between 90 and 150 ppm, and oxygen-bonded carbons observed between 170 and 180 ppm. The shift at 181.4 ppm is due to the amide bond. The signal at 172.90 ppm was assigned to the carbonyl carbons of carboxyl groups. Also, C19 should appear at 172 ppm. C15 and C5 should be found at 92–93 ppm, while C10 was observed at 112.33 ppm. Methyl and methylene carbons were observed at 20–23 ppm. ^13^C-NMR of PC is presented on Figure 3.

Other authors have observed similar shifts [39,40].

Several barriers exist to the systematic utilization of experiments, an essential stage in discovering new drugs. A few examples are the necessity to minimize animal experimentation, the quantitative constraints of tissue samples, and the prevalence of a compound. Given this, it is reasonable to believe that in silico computer models, which serve as both a useful addition and a workable replacement for biological investigations, may enhance or completely replace biological investigations [41]. That was the reason for applying several software items in our current study.

### 2.2. In Silico Assessment

Leveraging advanced tools such as the Quantitative Structure–Activity Relationship (QSAR) Toolbox (https://qsartoolbox.org/ accessed on 28 March 2024) [42], PreADME/T (https://preadmet.qsarhub.com/adme/, accessed on 17 April 2024), and SwissADME (http://www.swissadme.ch/index.php, accessed on 5 May 2024) [41], our study aimed to unravel the molecular intricacies of PC. The diverse computational profilers and models employed provided insights into its toxicity, physico-chemical properties, oral availability, drug-likeness, and potential biological targets. Table 1 provides an overview of the used in silico models.

The QSAR Toolbox, PreADME/T, and SwissADME web tools (http://www.swissadme.ch/ accessed on 17 April 2024)were selected for their comprehensive capabilities in evaluating compound safety and efficacy. The QSAR Toolbox is instrumental for its detailed analysis of metabolism and interactions with DNA and proteins, which are essential for assessing potential genotoxicity and mutagenicity. It provides critical data on structural alerts and metabolic pathways, ensuring a thorough evaluation of safety.

#### 2.2.1. Density Functional Theory (DFT) Approach

**Figure 4:**
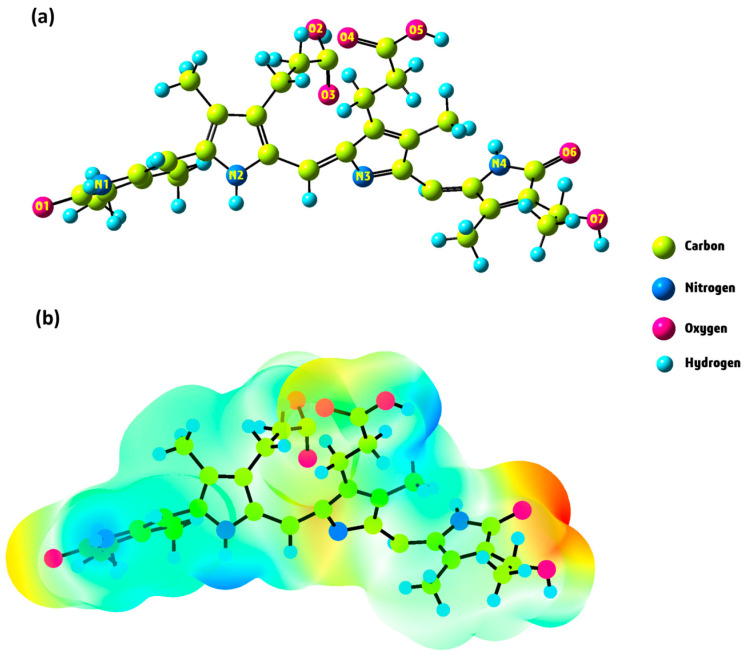
Optimized structure (**a**) and MEP surface (**b**) of PC molecule under DFT calculation.

The DFT/B3LYP/6–311G(d,p) [43,44] level of theory was utilized to optimize the structure of phycocyanin (PC). We used water as a solvent for the PC molecule by applying the CPCM model in the gas phase. Figure 4a illustrates the optimized structure of this compound in its lowest energy state. The electronic energy and dipole moment values for PC are determined as −2025.35 Hartree and 9.68 Debye, respectively. The high value of the dipole moment indicates that a notable dipole–dipole interaction occurs in the PC molecule [45].

The molecular electrostatic potential (MEP) map is an essential tool for visually representing changes in charge distribution within a compound. This map assists in pinpointing locations susceptible to electrophilic and nucleophilic attacks, as well as spotting hydrogen bonding [46]. The different colors, ranging from red to blue, depict the electrostatic potential values. On the MEP map, the red portion signifies a negative electrostatic potential, the blue region denotes a positive electrostatic potential, and the green zone corresponds to a neutral electrostatic potential [47]. Red and blue areas correspond to electrophilic and nucleophilic attacks, respectively. To obtain crucial information about the nucleophilic and electrophilic regions of the PC molecule, we generated the MEP surface using the DFT/B3LYP/6–311G(d,p) method, as depicted in Figure 4b. Analyzing the MEP map in Figure 4b, it exhibits a surface with positive potential (illustrated in blue) mainly around the hydrogens attached to N1, N2, N4, O2, O5, and O7 (namely, H_-N1_, H_-N2_, H_-N4_, H_-O2_, H_-O5_, and H_-O7_), indicating suitability for nucleophilic attack. In contrast, negative areas with red color are located around the O1, O3, O4, and O6 atoms, acting as binding locations for electrophilic attack. The molecular electrostatic potential values for the PC molecule range from −1.30 to +1.30 a.u.

**Figure 5:**
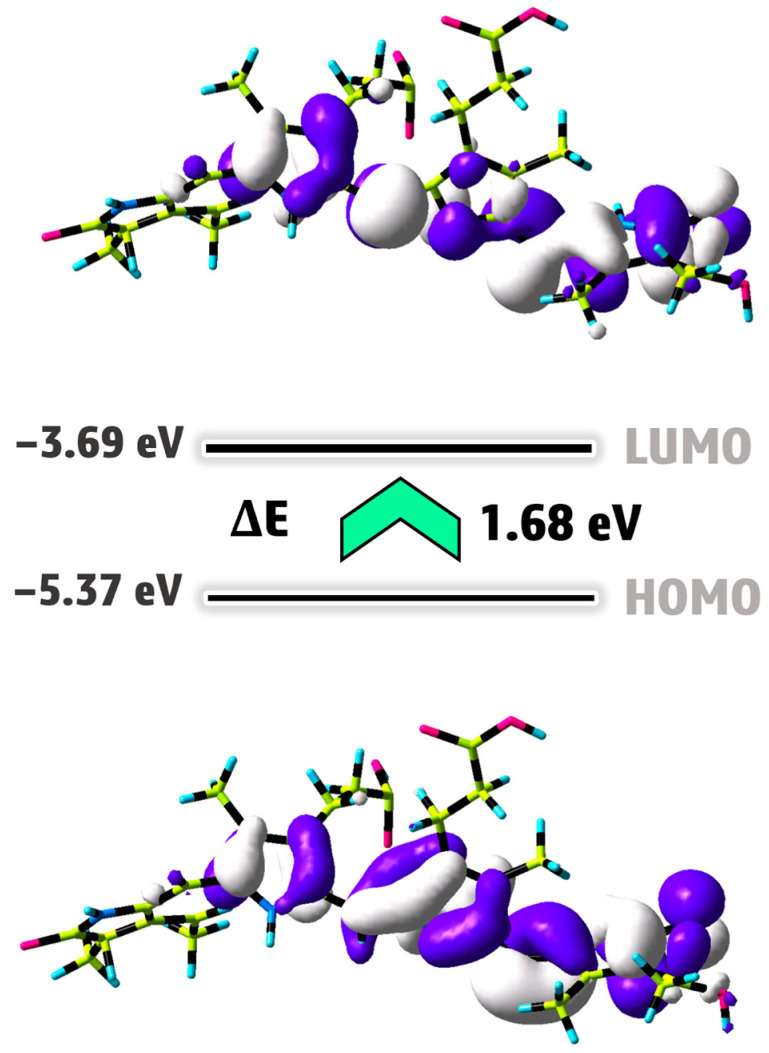
HOMO and LUMO surfaces of PC molecule obtained from DFT calculation.

The frontier molecular orbitals (FMOs) and their energy gap can be employed to thoroughly characterize the chemical reactivity, biological activity, and stability of the newly synthesized compound [48]. These orbitals, referred to as HOMO (the highest occupied molecular orbital) and LUMO (the lowest unoccupied molecular orbital), act as dependable markers for discerning the physical properties of compounds. Furthermore, the FMOs offer crucial understanding regarding interactions with biological receptors and other substances [49]. A small energy gap between HOMO and LUMO orbitals (∆E) signals significant chemical reactivity and biological activity [50]. Figure 5 displays the HOMO and LUMO orbitals of the PC molecule calculated using the DFT/B3LYP/6–311G(d,p) methodology. This figure shows that the electron density distributions on the PC molecule are the same at the HOMO and LUMO levels. The HOMO energy of the PC compound is determined to be −5.37 eV, owing to the presence of the π-orbital within the molecule, whereas the LUMO energy stems from the π∗-orbital at approximately −3.69 eV [51]. The PC molecule exhibits a low ΔE value, suggesting the electron transition from the π to π∗ orbital (HOMO→LUMO transition). Generally, the PC compound possesses a small value of ΔE (1.68 eV), which exhibits heightened chemical reactivity and biological activity. 

DFT calculations have been employed to establish the various quantum chemical characteristics of the PC compound, such as its absolute electronegativity (χ = − (E_HOMO_ + E_LUMO_)/2), absolute softness (σ = 1/η), global electrophilicity (ω = P_i_^2^/2η), absolute hardness (η = (E_LUMO_ − E_HOMO_)/2), and chemical potential (P_i_ = −χ) [52].

The values of σ, χ, ω, η, and Pi for PC are obtained to be 1.19 eV^−1^, 4.53, 12.21, 0.84, and −4.53 eV, respectively. The global electrophilicity component demonstrates how well electron acceptors can acquire extra electronic charge from the system. The high value of ω for PC suggests that this compound has the potential to engage in a greater variety of binding modes with macromolecules, such as protein receptors. The observed negative P_i_ parameter of the studied compound indicates that its structure remains intact, does not break down into basic elements, and is stable [53].

#### 2.2.2. QSAR Toolbox

Applying QSAR tools, including the in vivo rat metabolism simulator, Rat liver S9 metabolism simulator, and skin metabolism simulator, yielded crucial insights into the potential interactions of PC metabolites with DNA. Intriguingly, the parent structure showed no evidence of binding to DNA or proteins. This initial observation sets a foundation for a more detailed exploration of the metabolites.

An overview of the in silico tools used, along with their basic concepts and outputs, is presented in Table 2.

The in silico tools were selected based on their ability to simulate relevant biological environments and predict metabolite formation accurately. Other tools were considered; however, the selected tools offered a more comprehensive assessment of PC’s metabolism and potential interactions with DNA and proteins.

#### In Vivo Rat Metabolism Simulator

The in vivo rat metabolism simulator predicted the formation of four metabolites, presented in Table 3, and none exhibited binding to DNA or proteins. This suggests a favorable safety profile for these metabolites regarding genetic and protein interactions.

#### Rat Liver S9 Metabolism Simulator

Similarly, the Rat liver S9 metabolism simulator predicted the generation of two metabolites, presented in Table 4, both devoid of binding to DNA or proteins. This alignment in results across simulators reinforces the notion of a lack of DNA or protein reactivity in the identified metabolites.

#### Skin Metabolism Simulator

The skin metabolism simulator predicted eight metabolites, presented in Table 5, revealing a nuanced scenario. Four of these metabolites demonstrated structural alerts for epoxides, aziridines, thiiranes, and oxetanes associated with DNA binding, with mechanistic alerts indicating alkylation and direct-acting epoxides and related processes, as shown in Table 6. This raises concern regarding potential genotoxicity and necessitates a cautious evaluation of the risk associated with these specific metabolites.

Conversely, the remaining four metabolites exhibited no structural alerts, suggesting a lower likelihood of DNA binding. The divergent outcomes among the skin metabolites underscore the importance of assessing individual metabolites rather than generalizing toxicity predictions.

Two metabolites, identified through the skin metabolism simulator, exhibited structural alerts for epoxides, aziridines, and sulfuranes, with a mechanistic alert indicating a ring opening S_N_^2^ reaction, as presented in Table 7. These alerts signify the potential for protein binding, necessitating a focused discussion on the implications of these specific structural features.

The QSAR analysis of PC and its metabolites presents encouraging results for safety, with the parent structure and most metabolites showing no binding to DNA or proteins. However, identifying metabolites with structural alerts for genotoxicity and protein binding, particularly involving epoxides, requires careful consideration. Future studies and in vitro experiments are recommended for a deeper understanding and experimental validation.

For oral dosage forms emphasizing systemic exposure, the favorable QSAR results provide reassurance regarding potential genetic and protein interactions. Considering its safety profile, this supports the use of PC in oral formulations. However, for dermal applications, attention is needed for metabolites exhibiting structural alerts, ensuring safety aligns with the intended use.

#### 2.2.3. PreADME/T

The PreADME/T analysis of PC unveiled favorable outcomes concerning absorption, distribution, metabolism, and excretion, as detailed in Table 8. These findings provide valuable insights into the compound’s pharmacokinetic profile.

PC, derived from natural sources, exhibits favorable pharmacokinetic attributes. Its excellent absorption, indicated by a Human Intestinal Absorption (HIA) value exceeding 70%, suggests a high potential for effective gastrointestinal absorption, which is vital for optimizing bioavailability. The medium permeability in the Caco-2 cell model supports efficient absorption across the intestinal epithelium, aligning with low skin permeability and reducing the risk of systemic absorption for topical formulations.

The distribution analysis highlights weak plasma protein binding (87%), indicating a moderate affinity for plasma proteins. The intermediate CNS absorption, close to low CNS absorption, suggests a cautious interpretation of blood–brain barrier permeability. This distribution profile, with considerations for neuroactive effects, guides formulation strategies.

Metabolism assessment identifies PC as an inhibitor for CYP2C9 and a dual player (inhibitor and substrate) with CYP3A4. These interactions have implications for drug interactions, emphasizing the need for careful consideration in therapeutic formulations. The findings align with QSAR results, offering a holistic understanding of formulation strategies.

Further research, including in vitro assays and computational models, is essential for a nuanced understanding of safety profiles across diverse contexts and refining formulation strategies. This comprehensive approach ensures the optimal utilization of PC in various therapeutic and dietary applications.

#### 2.2.4. SwissADME—Lipinski’Rule of Five

The results obtained from the SwissADME analysis provide valuable insights into the drug-likeness of PC, a compound of interest for potential therapeutic applications. The computed molecular weight of 586.68 g mol^−1^ slightly exceeds Lipinski’s Rule of Five threshold of 500 Da. While this places PC outside the conventional range, it is essential to note that this rule is a general guideline rather than a strict rule, and exceptions exist for various successful drugs [54]. According to the rule, at least two parameters from four basic pharmacokinetic properties (MW ≤ 500, XLOGP3 ≤ 5, the number of hydrogen bond donors ≤ 5, and hydrogen bond acceptors ≤ 10.6) should be fulfilled for drug candidates. In terms of bioavailability (BA) [55], an optimal range of distinct properties have been reported, involving lipophilicity (XLOGP3: −0.7 to +5.0), size (MW: 150 to 500 g mol^−1^), polarity (TPSA: 20 to 130 Å2), ESOL or estimated solubility (log S: not more than 6), saturation (Fraction Csp3 or fraction of carbons in the sp3 hybridization: not less than 0.25), and flexibility (RB: no more than 9).

With a molecular weight of 586.68 g mol^−1^, the calculated TPSA value of 181.18 Å2, log Po/w (WLOGP) of 2.72, ten rotatable bonds, six hydrogen bond donors, and eight hydrogen-bond acceptors, PC is predicted to have low gastrointestinal absorption and BBB penetration. The analysis indicates the presence of seven hydrogen bond acceptors, which surpasses the recommended limit of ten according to Lipinski’s Rule of Five. Additionally, the compound possesses six hydrogen bond donors, well within the acceptable range of five. These findings suggest a moderately high potential for hydrogen bonding interactions, influencing the compound’s solubility and interactions within biological systems. The iLOGP value, an indicator of lipophilicity, is calculated at 3.75. While this surpasses Lipinski’s threshold of 5, it falls within a reasonable range for oral bioavailability.

Regarding the logP values, it is important to address that different models can produce varying results. For instance, the WLOGP value of 2.72 is calculated using one model, while other models might yield different logP values due to variations in their underlying algorithms and assumptions. The iLOGP value, an indicator of lipophilicity, is calculated at 3.75. Although this surpasses Lipinski’s threshold of 5, it falls within a reasonable range for oral bioavailability. The balance between hydrophilicity and lipophilicity is crucial for a compound’s absorption and distribution.

In drug-likeness, the slightly elevated molecular weight and the number of hy-drogen bond acceptors may raise considerations. However, these results should be in-terpreted cautiously, recognizing that exceptions exist for various successful drugs outside Lipinski’s conventional limits. The moderate iLOGP value suggests a balance between hydrophilicity and lipophilicity, potentially contributing to favorable oral bi-oavailability. Further discussion on the variability in logP models and their impact on bioavailability is essential to fully understand the relevance of these parameters.

#### 2.2.5. SwissTargetPrediction

SwissTargetPrediction provides valuable insights into the potential targets of PC, as presented in Table 9, revealing a diverse array of interactions. Several targets stand out, each with significant implications for understanding the compound’s pharmacological effects.

The target prediction analysis reveals a spectrum of potential targets for PC, each implicated in various cellular processes, supporting its recognized antioxidant, anti-inflammatory, neuroprotective, and immunomodulatory properties. The identified targets include ITGB1, ITGA4, ITGB7, ITGA4, ITGAV, ITGB3, ITGA2B, and ITGB3, which collectively suggest a role in modulating cellular adhesion and platelet aggregation. These interactions align with potential anti-thrombotic effects, emphasizing PC’s potential in cardiovascular health. Targets ALOX5 and PTGES indicate an interference with the arachidonic acid pathway, contributing to the anti-inflammatory activities observed in previous studies. The interaction with FKBP1A suggests potential immunomodulation, adding a layer to PC’s immune-regulatory effects. Furthermore, the interaction with MME implies neuroprotective effects, aligning with the compound’s potential in neurodegenerative conditions. Interactions with P2RY12 and EDNRA point toward cardiovascular benefits, anti-thrombotic effects, and anti-inflammatory properties. The engagement with LCK indicates a potential role in immune response regulation, complemented by interactions with TNF and PLA2G4B, emphasizing anti-inflammatory effects. The identified interactions with CCKBR suggest potential gastrointestinal influence, expanding the spectrum of PC’s physiological impact. Moreover, interactions with PTPN1 and PTPN2 imply the modulation of immune responses. These diverse interactions collectively underscore the multi-faceted therapeutic potential of PC, emphasizing the need for further experimental validation to elucidate the precise molecular mechanisms underlying its antioxidant, anti-inflammatory, neuroprotective, and immunomodulatory effects in specific biological contexts.

The identical probabilities reported for each target in Table 9 are attributed to the methodology used by the SwissTargetPrediction software. This tool employs a ligand-based approach, which evaluates the chemical similarity of the query compound to known ligands of potential targets. When compounds share structural features with multiple targets, the software might assign similar baseline probabilities. Additionally, the limitations of the tool in distinguishing between closely related binding sites can lead to uniform predictions.

The low probabilities reported in Table 9 from the SwissTargetPrediction software (http://www.swisstargetprediction.ch/ accessed on 5 May 2024) reflect the limitations of the tool’s ligand-based approach, which provide only an initial screening of potential targets. While the probabilities are below 0.067, they can still indicate potential interactions that warrant further investigation, particularly if these targets are relevant to the compound’s intended therapeutic effects.

The ProToxII web tool calculated PC’s LD50 as 7000 mg kg^−1^ and the compound’s toxicity class 6, which indicated the compound as practically non-toxic; still, a higher dosage of PC can cause respiratory and nutritional toxicity.

These results indicate that PC showed a good pharmacokinetic profile, and after additional tests it can serve as a new therapeutic agent.

## 3. Materials and Methods

### 3.1. Extraction of PC

This was performed as previously described by using “green methods” [56]. The highest yield of 14.88 mg g^−1^ with a purity index of 1.60 was achieved at a temperature of 40 °C for one hour and an ultrasonic wave frequency of 40 kHz. The extract was purified and lyophilized.

### 3.2. FTIR Spectroscopy

IR spectra were determined in KBr tablets on a VERTEX 70 FT-IR spectrometer (Bruker Optics, Ettlingen, Germany) in the wavelength range of 4000–400 cm^−1^ after 100 scans at a resolution of 2 cm^−1^.

### 3.3. NMR Spectroscopy

^13^C-NMR spectra were obtained using a Bruker Avance III HD 500 spectrometer (Bruker, Billerica, MA, USA) operating at a frequency of 126 MHz, respectively. A PC sample was dissolved in 99.95% D_2_O (20 mg/0.6 mL). Chemical shifts are given in relative ppm and were referenced to tetramethylsilane (TMS) (δ = 0.00 ppm) as an internal standard; the coupling constants are indicated in Hz. The NMR spectra were recorded at room temperature (ac. 295 K). MestreNova software (version 6.0.2-5475) was used in NMR spectra interpretation.

### 3.4. In Silico Toxicity Assessment

In silico toxicity assessment employs computational methods to predict the potential toxic effects of substances, relying on algorithms and simulations for analysis.

Compound: phycocyanin, known for diverse biological activities, was analyzed using computational tools.

#### 3.4.1. QSAR Toolbox

The Quantitative Structure–Activity Relationship (QSAR) Toolbox version 4.5 was employed for toxicity prediction. Various profilers, including the in vivo rat, Rat liver S9 and skin metabolism simulators, and DNA and Protein binding by OASIS, were utilized to assess potential toxicological endpoints and provide a comprehensive understanding of PC’s molecular characteristics [42].

#### 3.4.2. PreADME/T

PreADME/T software (https://preadmet.qsarhub.com/adme/, accessed on 27 April 2024) evaluated drug-likeness and oral availability parameters. Lipinski’s Rule of 5, a fundamental guideline in medicinal chemistry, was applied through PreADME/T to gauge PC’s adherence to fundamental physicochemical properties indicative of favorable oral bioavailability. This tool is also essential for predicting and optimizing PC’s absorption, distribution, metabolism, and excretion (ADME) properties, specifically focusing on its oral bioavailability [57].

#### 3.4.3. SwissADME

The online platform SwissADME (http://www.swissadme.ch/ accessed on 5 May 2024) assessed molecular weight, hydrogen bond acceptors, hydrogen bond donors, and log P_o/w_ (iLOGP) against Lipinski’s Rule of Five. Values were compared to Lipinski’s benchmarks: molecular weight < 500 Da, HB acceptors ≤ 10, HB donors ≤ 5, log Po/w (iLOGP) ≤ 5, evaluating PC’s drug-likeness [58].

SwissADME software (http://www.swisstargetprediction.ch/ accessed on 5 May 2024) was utilized for target prediction (in *Homo sapiens*), offering valuable insights into potential biological targets of PC. This computational tool analyzes pharmacokinetic properties and predicts the likelihood of the compound interacting with specific protein targets, aiding in its potential therapeutic effects [41].

#### 3.4.4. PASS Online Predictions

A computer-based program, PASS online (Prediction of Activity Spectra for Substances), was used to screen the biological activity of the compounds. The program predicts several thousand different biological activities based on the structural formula of a drug-like organic compound [41]. PASS has been used by many scientists for the discovery of new pharmaceutical agents in different therapeutic fields [59,60].

#### 3.4.5. Theoretical Prediction of Toxicity

For predicting the acute as well as organ toxicity of the compounds, the ProToxII free web tool was used. It predicts various toxicity endpoints, including acute toxicity and organ toxicities such as hepatotoxicity, cytotoxicity, carcinogenicity, mutagenicity, immunotoxicity, and toxicity targets. Toxicity class and LD50 values were also estimated [61,62].

#### 3.4.6. DFT Approach

Computational techniques were employed to explore the electronic structural parameters and behavior of the synthesized compound. DFT calculations were performed using the B3LYP hybrid functionals, assisted by the 6-311G(d,p) basis set, which accounted for all elements (carbon, oxygen, nitrogen, and hydrogen). The geometry of the compound was optimized using GaussView 6, along with the Gaussian 09W package [63]. All theoretical methods were conducted for the ground state in a gaseous phase without applying any limitations on symmetry. Furthermore, we used water as a solvent for the PC molecule using the CPCM model. To make sure that the harmonic frequencies were in real minima, frequency analysis had been investigated [64].

The molecular electrostatic potential (MEP) map and the molecular orbitals theory (specifically, the HOMO-LUMO surface) were examined to identify reactive regions and assess the molecule’s stability and chemical reactivity.

## 4. Conclusions

The current study provided additional structure–activity and structure–cytotoxicity information for the PC complex family. Indeed, this study demonstrates that the easy regulation of the nature of simple coordination to non-toxic transition metals results in compounds with high activity and low cytotoxicity of PC as a parent and clinical standard drug. DFT calculations were performed in order to predict the electronic behavior, HOMO-LUMO energy surface, and other quantum chemical reactivity parameters of the PC molecule. Furthermore, DFT confirmed the chemical reactivity and biological activity of the PC compound based on the energy gap. The theoretical results predicted by using DFT-based reactivity indexes correspond well with the experimental outcomes.

PC, derived from natural sources like cyanobacteria and algae, demonstrates promising pharmacokinetic attributes. The in silico toxicity assessment, leveraging advanced tools such as QSAR, PreADME/T, and SwissADME, offers valuable insights into its safety profile, bioavailability, metabolism, and potential biological targets. QSAR analysis indicates a favorable safety profile, with the parent structure and most metabolites showing no binding to DNA or proteins. However, the identification of metabolites with structural alerts necessitates careful consideration and calls for further in vitro studies.

The PreADME/T analysis highlights its low skin permeability, excellent intestinal absorption, and medium permeability, supporting its potential for oral administration. Distribution analysis suggests moderate plasma protein binding and a cautious interpretation of blood–brain barrier permeability, guiding formulation strategies. The metabolism assessment reveals interactions with key cytochrome P450 enzymes, influencing drug interactions and reinforcing QSAR findings. SwissADME identifies potential drug-likeness, supporting further exploration for pharmaceutical applications.

The target prediction analysis unveils a spectrum of potential targets, indicating PC’s involvement in cellular adhesion, anti-thrombotic effects, interference with the arachidonic acid pathway, anti-inflammatory activities, immunomodulation, neuroprotection, and cardiovascular benefits. These interactions collectively underscore the compound’s multi-faceted therapeutic potential.

However, further experimental validation is crucial to elucidate its precise molecular mechanisms and ensure safe and effective utilization in therapeutic and dietary contexts. This comprehensive approach, integrating in silico assessments and experimental studies, contributes to a nuanced understanding of PC’s pharmacological profile and informs future research directions.

## Figures and Tables

**Table 1:** Overview of QSAR Toolbox, PreADME/T and SwissADME.

Tool	Basic Concept	Type of Information Provided	Reason for Selection
QSAR Toolbox	A comprehensive tool for predicting the toxicity and metabolism of chemicals using various profilers and simulators.	Predictions of metabolic pathways, toxicity, protein and DNA interactions.	Integrates multiple profilers to provide a holistic view of a compound’s safety profile, including metabolism and toxicity.
PreADME/T	Focuses on predicting pharmacokinetic properties such as absorption, distribution, metabolism and excretion (ADME).	Pharmacokinetic parameters: intestinal absorption, plasma protein binding, BBB penetration, CYP inhibition, excretion, toxicity.	Provides insights into a compound’s ADME properties, critical for assessing bioavailability and potential drug interactions.
SwissADME	Analyzes drug-likeness based on Lipinski’s Rule of Five and other parameters; evaluates absorption, distribution, metabolism and excretion (ADME) and predicts the probability of the tested compound to bind to targets.	Drug-likeness parameters: molecular weight, lipophilicity (iLOGP), hydrogen bond donors/acceptors, TPSA, solubility. Target Prediction: likelihood of interaction with specific biological targets.	Assesses a compound’s suitability as a drug based on established pharmacokinetic and drug-likeness rules. Additionally, provides insights into potential biological targets, helping to understand the compound’s broader pharmacological effects.

**Table 2:** Overview of in silico tools used in analysis.

Tool	Basic Concept	Type of Information Provided	Reason for Selection
In vivo Rat Metabolism Simulator	Simulates metabolic processes in rats using 565 biotransformation reactions, includes phase I and II transformations.	Predictions of metabolic pathways and potential activation reactions.	Represents in vivo-like metabolism; crucial for understanding potential metabolic activation of xenobiotics.
Rat liver S9 Metabolism Simulator	Simulates liver-specific metabolism using a set of 565 biotransformation reactions from rodent liver microsomes and S9 fraction.	Prediction of liver metabolites and associated metabolic pathways.	Provides insights into liver metabolism and potential in vitro genotoxic effects.
Skin Metabolism Simulator	Simulates metabolism in the skin, including both rate-determining and non-rate-determining transformations.	Predictions of skin metabolites and their potential transformations.	Essential for evaluating dermal exposure and safety, given the differences between skin and liver metabolism.
DNA Binding by OASIS	Based on the Ames Mutagenicity model, it evaluates chemical structures for 117 structural alerts related to DNA interaction.	Identification of structural alerts and mechanistic domains related to DNA binding.	Focuses on predicting DNA mutagenicity; critical for assessing potential genotoxicity.
Protein Binding by OASIS	Analyzes chemical structures to identify potential interactions with proteins based on 112 structural alerts and 11 mechanistic domains.	Identification of structural and mechanistic alerts related to protein binding.	Provides comprehensive protein-binding alerts developed by industry consortia; essential for assessing potential protein interactions.

**Table 3:** Numbers and structures of predicted metabolites of PC obtained after in vivo rat metabolism simulator.

Structure and Metabolite Number	
**1**	**2**
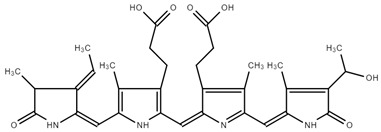	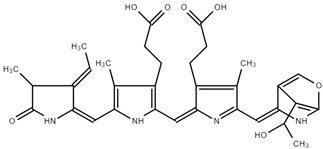
**3**	**4**
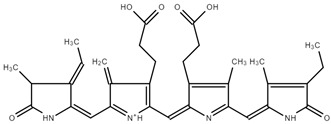	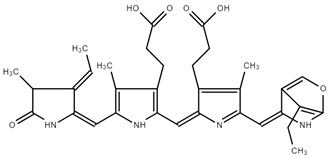

**Table 4:** Structure and metabolite number of predicted metabolites of PC obtained after Rat liver S9 metabolism simulator.

Structure and Metabolite Number	
**1**	**2**
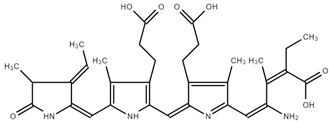	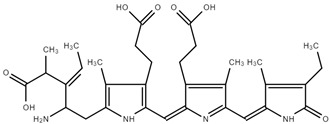

**Table 5:** Structure and metabolite number of predicted metabolites of PC obtained after skin metabolism simulator.

Structure and Metabolite Number	
**1**	**2**
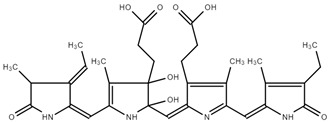	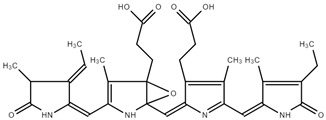
**3**	**4**
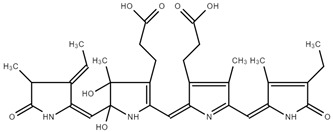	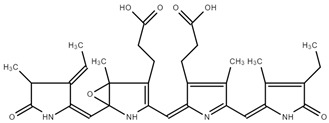
**5**	**6**
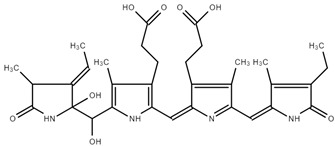	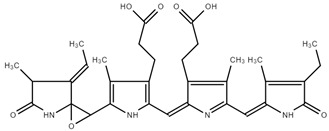
**7**	**8**
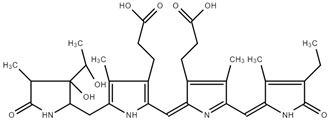	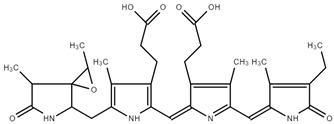

**Table 6:** Binding of PC metabolites to DNA.

Metabolite Number	Structural Alert	Mechanistic Alert	Mechanistic Domain
1, 3, 5, 7	No alert found	-	-
2, 4, 6, 8	Epoxides, aziridines, thiiranes, and oxetanes	Alkylation, direct-acting epoxides and related	S_N_^2^

**Table 7:** Binding of PC metabolites to proteins.

Metabolite Number	Structural Alert	Mechanistic Alert	Mechanistic Domain
1–5, 7	No alert found	-	-
6, 8	Epoxides, aziridines, and sulfuranes	Ring opening S_N_^2^ reaction	S_N_^2^

**Table 8:** Pharmacokinetic and toxicological parameters of PC predicted using PredADME/T web tool.

PreADME/Tox Parameters				
**Absorbtion**				
Human Intestinal Absorption		Caco-2		Skin Permeability
84.638039		20.3983 nm/s		−3.80287 logKp, cm/h
**Distribution**				
Plasma Protein Binding			Blood–Brain Barrier	
87.494840			0.19048	
**Metabolism**				
CYP2C19	CYP2C9		CYP2D6	CYP3A4
-	Inhibitor		-	Inhibitor/Substrate
**Excretion**				
MDCK				
0.0434166 nm/s				
**Toxicity**				
Ames Test		Carcinogenicity Mouse		Carcinogenicity Rat
Mutagen		Negative		Positive

Human intestinal absorption (HIA): low absorption 0.00–20.00%; moderate absorption 20.00–70.00%; excellent absorption 70.00–100.00%. Caco-2 cell permeability: high permeability > 70.0 nm/s; medium permeability 4.0–70.0 nm/s; low permeability < 4.0 nm/s. Skin permeability: values vary from −4.00 to 6.00 logKp, cm/h. Plasma Protein Binding (PPB): strong connection > 90.0%; weak connection < 90.0%. Blood–Brain Barrier (BBB): high CNS absorption > 2.00; intermediate CNS absorption 0.10 ÷ 2.00; low CNS absorption < 0.10 The permeability of the BBB is assessed using the ratio *C*_brain_/*C*_blood_, where *C*_brain_ and *C*_blood_ represent the concentrations of the drug in the brain and blood, respectively. Therefore it is dimensionless. MDCK: low permeability < −1.0 nm/s; medium permeability −1.0 ÷ 1.0 nm/s; high permeability > 1.0 nm/s. Ames test: positive: mutagenic; negative: non-mutagenic. Carcinogenicity: positive: carcinogenic; negative: non-carcinogenic.

**Table 9:** Target prediction for PC using SwissTargetPrediction web tool.

Target	Common Name	Target Class	Probability
Integrin alpha-4/beta-1	ITGB1 ITGA4	Membrane receptor	0.06613
Integrin alpha-4/beta-7	ITGB7 ITGA4	Membrane receptor	0.06613
Integrin alpha-V/beta-3	ITGAV ITGB3	Membrane receptor	0.06613
Integrin alpha-IIb/beta-3	ITGA2B ITGB3	Membrane receptor	0.06613
Arachidonate 5-lipoxygenase	ALOX5	Oxidoreductase	0.06613
Prostaglandin E synthase	PTGES	Enzyme	0.06613
FK506-binding protein 1A	FKBP1A	Isomerase	0.06613
Neprilysin (by homology)	MME	Protease	0.06613
Purinergic receptor P2Y12	P2RY12	Family A G proteincoupled receptor	0.06613
Endothelin receptor ET-A	EDNRA	Family A G proteincoupled receptor	0.06613
Tyrosine-protein kinase LCK	LCK	Kinase	0.06613
Glutamate carboxypeptidase II	FOLH1	Protease	0.06613
TNF-alpha	TNF	Secreted protein	0.06613
Phospholipase A2 group 1VB	PLA2G4B	Enzyme	0.06613
Cholecystokinin B receptor	CCKBR	Family A G proteincoupled receptor	0.06613
Protein-tyrosine phosphatase 1B	PTPN1	Phosphatase	0.06613
T-cell proteintyrosine phosphatase	PTPN2	Phosphatase	0.06613

## Data Availability

The data presented in this study are available on request from the corresponding author.
